# Long-Lasting Impact of Maternal Immune Activation and Interaction With a Second Immune Challenge on Pig Behavior

**DOI:** 10.3389/fvets.2020.561151

**Published:** 2020-11-23

**Authors:** Haley E. Rymut, Courtni R. Bolt, Megan P. Caputo, Alexandra K. Houser, Adrienne M. Antonson, Jalisa D. Zimmerman, Maria B. Villamil, Bruce R. Southey, Laurie A. Rund, Rodney W. Johnson, Sandra L. Rodriguez-Zas

**Affiliations:** ^1^Department of Animal Sciences, University of Illinois at Urbana-Champaign, Urbana, IL, United States; ^2^Department of Crop Sciences, University of Illinois at Urbana-Champaign, Urbana, IL, United States; ^3^Neuroscience Program, University of Illinois at Urbana-Champaign, Urbana, IL, United States; ^4^C. Woese Institute for Genomic Biology, University of Illinois at Urbana-Champaign, Urbana, IL, United States; ^5^Department of Statistics, University of Illinois at Urbana-Champaign, Urbana, IL, United States

**Keywords:** porcine reproductive and respiratory syndrome, Poly(I:C), maternal immune activation, sickness behavior, locomotor

## Abstract

The combined effects on pig behavior of maternal immune challenge during gestation followed by a second immune challenge later in life have not been studied. Porcine reproductive and respiratory syndrome virus (PRRSV) infection during gestation can elicit maternal immune activation (MIA) yet the interactions with the offspring response to a second immune challenge after birth remains unexplored. Knowledge on the response to viral challenges in rodents has been gained through the use of the viral mimetic polyinosinic-polycytidylic acid (Poly(I:C)), yet the effects of this immune stimulant on pig behavior have not been assessed. This study advances the understanding of the combined effect of MIA and a second immune challenge later in life on female and male pig behavior. Three complementary experiments enabled the development of an effective Poly(I:C) challenge in pigs, and testing the interaction between PRRSV-elicited MIA, Poly(I:C) challenge at 60 days of age, and sex on behaviors. Individual-level observations on sickness, locomotor, and social behaviors were measured 1–3 h after Poly(I:C) challenge. Vomiting, panting, lethargy, walking, laying, playing, and touching behaviors were analyzed using generalized linear mixed effect models. Results indicated that a Poly(I:C) dose of 1 mg/kg within 1 h after injection increased the incidence of laying and sickness behavior. The Poly(I:C) challenge decreased the incidence of locomotor behaviors and activity levels. Pigs exposed to MIA had lower rates of social behaviors such as playing. The combined effect of PRRSV-elicited MIA and Poly(I:C) immune challenge further sensitized the pigs to behavior disruption across sexes including changes in sternal and lateral laying, walking, lethargy, and touching incidence. Notably, the effects of Poly(I:C) immune challenge alone on behaviors tended to be more extreme in males, whereas the effects of Poly(I:C) following MIA tended to be more extreme in females. Our findings demonstrate that MIA and Poly(I:C) affected behaviors, and the viral mimetic effects shortly after injection can offer insights into the prolonged effect of postnatal viral infections on feeding, social interactions and health status. Management practices that reduce the likelihood of gestational diseases and accommodate for behavioral disruptions in the offspring can minimize the impact of MIA.

## Introduction

Immune challenges experienced by the mother during gestation can activate the immune response, known as maternal immune activation (MIA) (1–3). The maternal inflammatory response to pathogen, psychological, or other insults can result in fetal exposure to maternal cytokines and stress hormones, leading to a disruption of fetal neurodevelopmental processes (4, 5). The association between maternal immune activation and behavior disorders including schizophrenia spectrum disorder (SSD) and autism spectrum disorder (ASD) have been established in humans and biomedical models (6, 7). Comparable effects of MIA on food production animals have been reported (1–3). In a study of the effects of MIA, pigs from gilts inoculated with porcine reproductive and respiratory syndrome virus (PRRSV) at gestation day (GD) 76 presented lower sociability and preference for social novelty than pigs from control gilts at 2 weeks of age (1–3). Similar disruption of social behaviors in response to MIA has been reported across species including rodent models of ASD-like and SSD-like symptoms (6, 8, 9).

The effect of MIA on offspring behaviors later in life is sex dependent. The prevalence and symptoms of MIA-associated behavior disorders as akin to those observed in ASD and SSD tend to be differentially prevalent between sexes (6, 7). In response to gestational stressors, some ASD-like behavioral disorders are more prevalent in young males (10, 11) whereas others such as SSD are more prevalent in females (12, 13). In a study of MIA using valproic acid to elicit ASD-like phenotypes, male rats displayed social behavior abnormalities, whereas female rats displayed heightened anxiety (14).

In addition to sex, the effects of MIA on the offspring immunophysiology and behavior depend on the exposure to a second immune challenge later in life and may potentiate the impact of MIA on the offspring. Studies using rodent and primate models of SSD, ASD and other behavioral disorders offered evidence of the combined effects of MIA and a second challenge (15–18). The double-hit hypothesis proposes that exposure to a first immune challenge during prenatal development (i.e., MIA) elicits long-term neural and immune disruptions that subsequently modify the offspring response to a second immune challenge later in life (19). Offspring exposed to MIA can be more sensitive or more tolerant to a second stress and this response can modulate the incidence of behavioral disorders (20).

Several types of immune challenges have been used to study the combined effects of MIA followed by a second immune challenge later in life. The viral mimetic agent polyinosinic-polycytidylic acid (Poly(I:C)), a synthetic analog of the double-stranded viral RNA, is administered for the stimulation of inflammatory response, including eliciting MIA in gestating rodents (21). Poly(I:C) binds to the toll-like receptor 3, and this reaction stimulates the production of pro-inflammatory cytokines including interleukin-1 beta, interleukin-6, and tumor necrosis factor alpha. This mode of action positions Poly(I:C) as an effective simulator of an acute phase response to viral infection (21). Poly(I:C) has a high degree of construct validity eliciting short-term and defined immune activation and sickness-like behaviors (22).

Poly(I:C) is used instead of a live virus or endotoxin because this viral mimetic does not require stringent biosafety precaution, allows for precise control of dose-immune response, and enables precise timing of activation. *In vivo* studies of Poly(I:C) in pigs has been mostly in the context of vaccine testing (23, 24). Two studies of Poly(I:C) administered to ~1-month-old pigs at a dose of 0.5 mg/kg body weight, profiled circulating metabolites and cytokines, but did not monitor behaviors (25, 26).

In this study, PRRSV was chosen to elicit MIA in pregnant gilts due to its ability to be used in a translational biomedical model and its impact on the swine industry. Viral diseases cause major financial losses to the swine industry worldwide, hindering reproductive and growth rate, and increasing the mortality rate and veterinary and management costs (27–29). The annual costs caused by PRRSV to the US swine industry are estimated to surpass $600 million (30, 31). Multiple sources of revenue loss, including lower reproductive and growth rate, and higher veterinary expenses have been identified on the infected pigs and on the offspring of infected pigs (32), and higher incidence of respiratory disorders of the pigs directly infected.

PRRSV infection of gilts and sows during gestation can impact the fetuses at the neurodevelopment, physiology, and behavior levels and further magnify the losses from the direct action of PRRSV on the female. The offspring from PRRSV-infected gilts that were exposed to a second challenge using the bacterial endotoxin lipopolysaccharide (LPS) at postnatal day (PD) 14 had significantly higher circulating levels of pro-inflammatory cytokines and cortisol than pigs from PRRSV-infected gilts that did not receive the second challenge (1). Additional studies are needed to understand the effects of a double-hit challenge on the behavior of older pigs.

The overarching goal of this study is to advance the understanding of the effect of the double-hit paradigm on the behavior of PD 60 pigs, shortly before transition from nursery to the growing and finishing phase. The combined effects on behavior (1) of MIA elicited by PRRSV, (2) of a viral mimetic challenge, and (3) the interactions of these effects with sex were tested. A supporting objective is to develop a Poly(I:C) model that elicits reliable behavioral response in pigs. Results from the longitudinal analysis of individual-level observations could guide management practices to ameliorate the effects of pre- and post-natal immune challenges on pig behaviors that could have deleterious effects on pig performance and health.

## Materials and Methods

The Institutional Animal Care and Use Committee at the University of Illinois approved the animal experiments (IACUC # 20026). Results from three complementary experiments addressing our multifactorial objective are presented. Experiment 1 enabled the identification of an effective Poly(I:C) dose and timeline protocol to elicit sickness behaviors across females and intact males. Experiment 2 aided in the characterization of both sex-dependent and independent effects of Poly(I:C) on pig behaviors. Lastly, Experiment 3 enabled us to test for interactions between PRRSV-elicited MIA, a second immune challenge using Poly(I:C), and sex effects on pig behaviors at PD 60. At 60 days of age, pigs are being transitioned from nursery to the growing and finishing phase and experience environmental changes. Understanding the effect of immune challenge during this stage while removing the management transition reduces the sources of variation that could contribute to behavior changes.

### Experiment 1. Study of the Effects of Poly(I:C) Dose and Time Course on Behaviors Across Sexes

Pigs from the University of Illinois Swine Research Center herd, from healthy PRRSV-free PIC Camborough® gilts artificially inseminated with PIC 359 boars (PIC, Hendersonville, TN) were studied. Pigs from separate litters remained with their gilts until weaning at approximately PD 21, and thereafter were housed in a standard growing pen. The pigs had *ad libitum* access to water, received a diet based on corn and soybean meal to meet the nutritional needs of growing pigs, and were selected among to be similar age and weight. The pen dimension allowed for a density of 1.1 m^2^/pig, and included feeder and nipple drinker, and had partly solid and partly slatted concrete floor. The pigs were of matching age and weight and were randomly assigned to receive one of three Poly(I:C) (Sigma, St. Louis, MO) treatment levels. Pigs from both sexes were administered an intraperitoneal (i.p.) injection of either 1.0 mg/kg of body weight of Poly(I:C), 0.5 mg/kg of Poly(I:C), or the comparable volume of sterile phosphate buffered saline (PBS) as a control at PD 60.

A trained technician recorded the behavior of the pigs. The observer remained outside the pen to minimize disruptions (33), and the pigs were identified using ear tags and markings. The behavior of individual pigs was recorded every 15 min across 3 h following published protocols (33, 34). Prior to the trial, the observer received training to detect behaviors in a consistent manner based on existing videos (34). During each scan, the identity of the pig and the occurrences of the locomotor and sickness behaviors were recorded. The behaviors were recorded for ~3 h after the Poly(I:C) or saline injections starting at 7:00 AM. Per behavior, in total 120 observations across 10 pigs (*n* = 4 Poly(I:C) 1 mg/kg, *n* = 4 Poly(I:C) 0.5 mg/kg, and *n* = 2 saline) were recorded. For the binary behavior response variables (lower information than continuous variables), power calculations were performed using the POWER procedure (SAS/STAT software, Version 9.4, 2019; SAS Institute, Cary, NC, USA). These calculations assumed a logistic regression model including the effect of Poly(I:C) level (control, 0.5, and 1 mg/kg) and the covariates. The statistical power was 0.61 to detect behavior probabilities between 0.3 and 0.7, at type I error rate = 0.05. This pilot experiment enables us to identify the safe dose of Poly(I:C) that could trigger behavior changes, and the time after challenge when the changes could be observed. Subsequent experiments enabled us to validate the results from this trial and expand the study to consider MIA effects.

The recorded behaviors encompassed the inactivity score and sickness behaviors of the pigs. Sickness behavior was defined as detection of any vomiting, diarrhea, panting, or shivering. The range of inactivity scores recorded varied from highly active (level 1) to inactive (level 8). The inactivity scoring, and corresponding definition included 1: running around, not playing or interacting with other pigs or environment; 2: running around, playing or interacting with other pigs or environment; 3: walking around, playing or interacting with other pigs or environment; 4: walking around, not playing or interacting with other pigs or environment; 5: sitting or standing, not moving; 6: lying down, awake; 7: lying down, asleep; and 8: lying down, shivering, unresponsive to environment.

### Experiment 2. Study of the Effects of Poly(I:C) Immune Challenge and Sex on Behaviors

A healthy and PRRSV-free PIC Camborough® gilt from the University of Illinois swine herd was artificially inseminated with semen from a PIC line 359 boar. At GD 69, the gilt was moved into a containment chamber, had *ad libitum* access to water, and received 2.3 kg of a standard corn-soybean meal-based gestation diet daily (1). Farrowing was induced on GD 113 using a 10 mg (2 mL) injection of dinoprost tromethamine Lutalyse® (Pfizer, New York, NY). The pigs remained with the sow in the farrowing crates until weaning at PD 21 (35), and were group-housed in two pens afterwards. The pigs had similar age and weight and were assigned at random to the pens and the pens only housed the pigs used in the trial. The pens were located in the Managed Animal Facilities at the Division of Animal Resources of the University of Illinois at Urbana-Champaign, and the dimensions of the rooms enabled a pig density of 1.15 m^2^/pig. The pens had a feeder and water dispenser, plastic slatted floors, and balls for enrichment. The pigs had *ad libitum* access to water, received a diet based on corn and soybean meal to meet the nutritional needs of growing pigs and both sexes and treatment groups were represented in each pen. At PD 60, half of the pigs received an i.p. injection of 1.0 mg/kg of Poly(I:C), and the remainder pigs served as controls were i.p injected with the comparable volume of sterile PBS.

The behavior of individual pigs was recorded with the video camera that was mounted above the pen. Each pig was videotaped at 30 frames per second in their pens. A continuous scan of 15 s every 3 min was annotated. All the scans were reviewed by the same trained technician at 30 frames per second and slow motion was used when needed (e.g., pigs in proximity or sudden movements). The technician who was blind to the treatment assignment and prior to the experiment, the technician received training to detect behaviors in a consistent manner (34, 36). During each scan, the identity of the pig and the occurrences of the locomotor and sickness behaviors were recorded. The behaviors were recorded starting at 7:00 a.m. Per behavior, in total 500 observations across 10 pigs (*n* = 6 Poly(I:C) 1 mg/kg; *n* = 4 saline) were recorded. The recorded behaviors included vomiting, diarrhea, panting, lethargy, walking, laying, playing, touching other pigs, drinking or eating, standing, and sitting. Behavior characterizations applied previously used definitions (37). Behavior definitions include, standing defined as standing with all hooves on the floor; sitting defined as sitting with hind quarters on the floor while remaining in an upright position; lateral or sternal laying defined as laying on the side or belly, respectively; walking defined as moving in a forward or backward direction; playing defined as gamboling or pivoting with another pig or with a toy; drinking defined as stand with mouth around the drinking spout; and eating defined as standing with head in the feeding box. The definition of touching encompassed the behaviors of nosing, nibbling, sucking or chewing any body part of a pen mate (37). Lethargy was recognized as a state of sluggishness, dullness, listlessness, with lack of activity or response to actions from other pigs in the pen (38). The body temperature of the pigs was recorded by rectal thermometer prior to and 1 h after Poly(I:C) injection.

### Experiment 3. Study of the Effects of Maternal PRRSV Activation, Poly(I:C) Immune Challenge, and Sex on Behaviors

In alignment with Experiment 2, 12 healthy and PRRSV-free PIC Camborough® gilts from the University of Illinois swine herd were artificially inseminated with semen from PIC line 359 boars across 2 replicates run 2.5 months apart. At GD 69 the gilts were moved into individual containment chambers with *ad libitum* access to water, and were fed daily 2.3 kg of a standard corn-soybean meal-based gestation diet (1). The gilt body temperature (recorded by rectal thermometer) and feed refusal were measured daily from GD 69 until farrowing. Following protocols proven to elicit MIA (1), on GD 76 half of the gilts were intranasally inoculated with live PRRSV strain P129-BV (School of Veterinary Medicine at Purdue University, West Lafayette, IN) using 5 mL of 1 × 10^5^ median tissue culture infectious dose (TCID_50_) diluted in sterile Dulbecco's modified Eagle medium (DMEM; 5 mL total volume). The remaining gilts that served as a control were intranasally inoculated with 5 ml of sterile DMEM. The PRRSV-treated gilts received the maximum daily feeding (2.3 kg of the standard diet), and the feed refusal of these gilts was measured. The average feed intake consumed by the PRRSV-treated gilts was provided to the Control gilts on the next day.

Farrowing conditions, post-weaning management, pen location and dimensions, and Poly(I:C) injection at PD 60 occurred as described in Experiment 2. Behaviors were recorded every 5 min up to 1 h after injection by a trained experimenter blind to the treatment assignment on group-housed pigs distributed across 10 pens (mixed sex and litter within pen). Measurements were recorded across four replicates starting daily at 7:00 a.m. All behaviors (with exception of vomiting and diarrhea) were observed across the four replicates and the observations were monitored on consecutive days.

A trained technician monitored the pig behaviors in the pens. The observer remained outside the pen to minimize disruptions (33). The behavior of individual pigs was recorded approximately every 4 min across 1 h following published protocols (33, 34). Prior to the trial, the observer received training to detect behaviors in a consistent manner based on exiting videos (34). The recorded behaviors were mutually exclusive. During each scan, the identity of the pig and the occurrences of the locomotor and sickness behaviors were recorded. Per behavior, 624 observations were recorded across 52 pigs (MIA group, Poly(I:C) group, and sex balanced, 4 (pigs) x13 (pens) were recorded. Consistent with Experiment 2, the recorded behaviors included vomiting, diarrhea, panting, shivering, lethargy, walking, laying, playing, touching, eating, standing, and sitting. Body temperature of the pigs was recorded by rectal thermometer immediately prior to and an hour after Poly(I:C) injection.

### Statistical Analysis

The individual pig behaviors were recorded post-injection and were analyzed as observed or not observed binary variables. The binary response variables were analyzed using a logistic mixed effects model (39). The inactivity level was analyzed using a Poisson mixed effects model. These generalized linear models included the fixed effects of Poly(I:C) treatment, sex, time at measurement, MIA, and all interactions. The levels of the Poly(I:C) treatment in Experiment 1 were 0 mg/kg (Saline), 0.5 mg/kg or 1 mg/kg of body weight; and in Experiments 2 and 3 the levels were 0 mg/kg (Saline), and 1 mg/kg of Poly(I:C). The levels of the MIA effect in Experiment 3 were PRRSV and Control. The time levels were hour 1, 2, or 3 in Experiment 1 and the more frequent measurements within the first hour in Experiments 2 and 3 were modeled using linear and quadratic trends in minutes. The focus of the present study was to characterize the peak behavior response to Poly(I:C) treatment, in consideration of MIA and sex effects. All pigs in a pen were monitored in consistent order.

Recognizing that particular clusters of observations were not independent, multiple model specifications were incorporated in the analysis. The studied gilts were selected at random from the gilts available in the herd and pigs were selected at random from the litters of individual gilts, supporting the modeling of gilts and the pig observations as random effects that follow a Normal distribution. The blocking random effects of gilt and pen and the covariate of body weight were included in the model. Heterogeneity of variance across random effect levels was assessed using the Bayesian Information Criterion. The correlation between the longitudinal observations measured within pig was accommodated using a repeated-measurements model specification. The autoregressive order 1 variance-covariance structure was supported by the data based on Bayesian Information Criterion. The detection of behavior modifications associated with handling required to inject Poly(I:C) or Saline were minimized by excluding from the analysis the initial 5 min immediately after the injection. The least square means (and standard errors) were estimated in the underlying logit scale, and were then back-transformed into behavior probabilities per treatment and sex groups. The analyses of the generalized linear mixed effect models were implemented using GLIMMIX procedure with the Kenward-Rogers adjustment of degrees of freedom (SAS/STAT software, Version 9.4, 2019; SAS Institute, Cary, NC, USA). The analytical partitioning of the Poly(I:C) effect within sex and MIA pig groups, and of the effect of MIA within Poly(I:C) and sex pig groups was implemented using the /SLICE option. The least square mean and contrast estimates, and associated standard errors for inactivity score are reported. For the binary behavior measurements, the least square mean and contrast estimates (and standard errors) were estimated in the underlying logit scale. The back-transformation of the previous estimates into behavior probabilities are reported by treatment and sex groups.

The effects of PRRSV-elicited MIA on body temperature and feed consumption of the gilts were analyzed using a linear mixed effects model. The model included the main effects of MIA, measurement day, all the interactions among the main effects, and the random effects of gilt and replicate. The change in the body temperature of the PD 60 pigs was analyzed using a linear mixed effects model. The model describing the difference in body temperature 1 h after relative to before the Poly(I:C) injection included the main effects of Poly(I:C) pig treatment, MIA, sex, interactions among the main effects, and the random effects of gilt and replicate. The analyses of the linear mixed effect models were implemented using the MIXED procedure with the Kenward-Rogers adjustment of degrees of freedom and Restricted Maximum Likelihood approach (SAS/STAT software, Version 9.4, 2019). The least square mean and contrast estimates, and associated standard errors for body temperature and feed consumption are reported.

## Results

###  Experiment 1. Effects of Poly(I:C) Dose and Time Course on Behaviors Across Sexes

**Figure 1:**
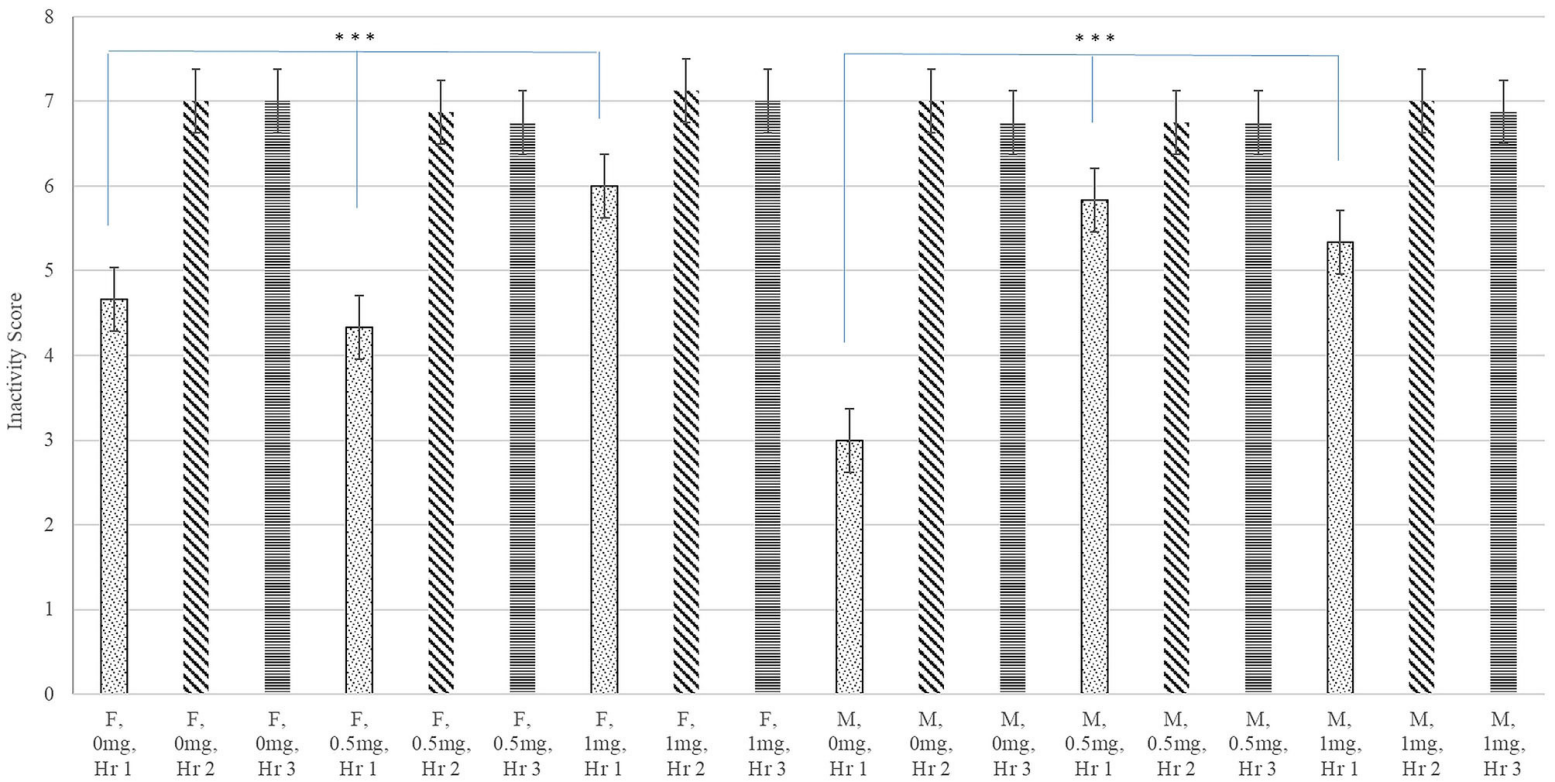
Inactivity score (estimate and standard error of the mean) by Poly(I:C) dose (0, 0.5, or 1 mg/kg of body weight), sex (F = female or M = male), and hour (Hr 1, 2, or 3) after injection. ***Differences between Poly(I:C) doses within sex in hour 1 significant at *P*-value < 0.005 (*n* = 10). Scores are unitless and values range from 1 (highly active) to 8 (inactive).

**Figure 2:**
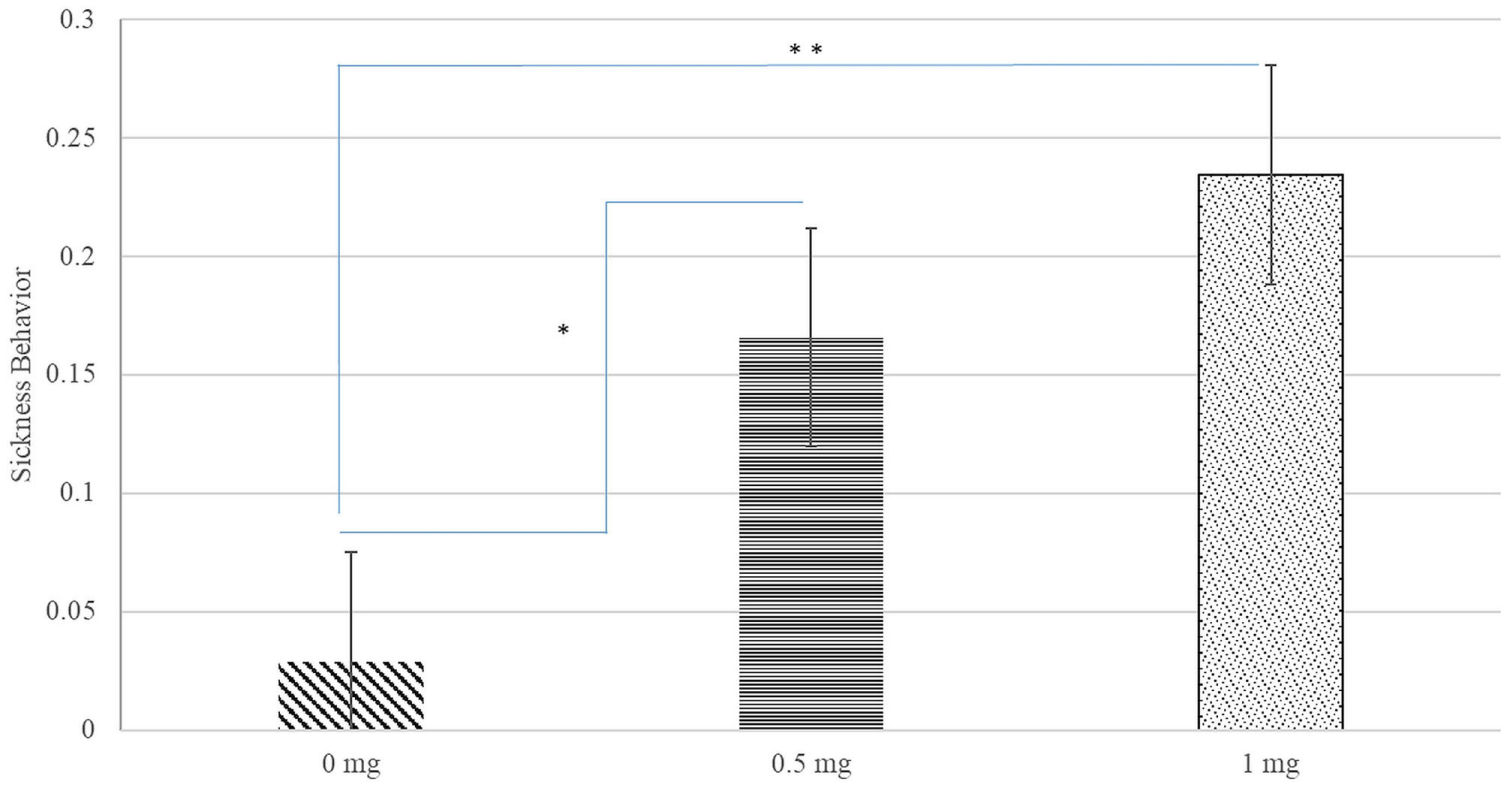
Probability of sickness behavior (estimate and standard error of the mean) by Poly(I:C) dose (0, 0.5, or 1 mg/kg of body weight). **, *Difference between Poly(I:C) doses significant at *P*-value < 0.005, and *P*-value < 0.05, respectively (*n* = 10). Probabilities are unitless and values range from 0 to 1.

A significant interaction between Poly(I:C) dose, sex, and hour after injection was identified for inactivity score (*P*-value < 0.020) and a significant Poly(I:C) dose effect was detected for the probability of sickness behavior (*P*-value < 0.019). Figure 1 depicts the patterns of inactivity score, an index of locomotor activity and sickness symptoms, across pig groups characterized by the Poly(I:C) dose-sex-hour categories. The inactivity score estimates were significantly higher in pigs injected 1.0 and 0.5 mg/kg of Poly(I:C) relative to pigs injected Saline. The most significant difference of inactivity between Poly(I:C) doses was detected in the first hour after injection for males (*P*-value < 0.001) and females (*P*-value < 0.004). Figure 2 depicts the probabilities of sickness behavior across pig groups characterized by the Poly(I:C) dose-sex-hour categories. While Saline-treated pigs did not exhibit sickness behaviors, the probability of sickness behavior was highest (Probability = 0.234) among pigs injected 1.0 mg/kg Poly(I:C), followed by pigs injected 0.5 mg/kg Poly(I:C) dose (Probability = 0.165).

### Experiment 2. Effects of Poly(I:C) Immune Challenge and Sex on Behaviors

Significant effects of Poly(I:C) treatment and treatment-by-sex interaction were identified in multiple activity and sickness behaviors during the first hour after Injection. Table 1 presents a summary across behaviors of the effects (*P*-values) of sex, treatment, and interaction, and of the effect of Poly(I:C) treatment within sex. Table 2 presents the estimated probabilities of individual behaviors (and associated standard error) by treatment and sex group.

**Table 1:** Statistical significance of the effects of pig immune treatment, sex, and interaction, and of the effect of treatment within sex, by behavior corresponding to Experiment 2.

**Behavior**	**Sex**	**Treatment^a^**	**Treatment × Sex**	**Treatment in females**	**Treatment in males**
Lateral laying	0.679^b^	0.031	0.275	0.026	0.152
Sternal laying	0.056	0.001	0.614	0.001	0.001
Total laying	0.001	0.090	0.815	0.213	0.109
Lethargy	0.039	0.008	0.494	0.072	0.007
Standing	0.002	0.239	0.535	0.611	0.106
Touching	0.407	0.316	0.703	0.322	0.516
Walking	0.716	0.032	0.140	0.251	0.011

a *Pig treatment levels: 1 mg/kg of Poly(I:C) or Saline*.

b *P-value (n = 10 pigs)*.

**Table 2:** Probability of behavior estimates (Est) and standard error (SE) by sex and pig immune treatment corresponding to Experiment 2.

	**Female**				**Male**				
	**Saline^a^**		**Poly(I:C)**		**Saline**		**Poly(I:C)**		**Effect^b^**
**Behavior**	**Est^c^**	**SE**	**Est**	**SE**	**Est**	**SE**	**Est**	**SE**	***P < 0.05***
Lateral laying	0.17^1^	0.08	0.01	0.01	0.01	0.07	0.01	0.01	Trt
Sternal laying	0.57	0.07	0.90	0.04	0.47	0.07	0.82	0.05	
Total laying	0.85	0.04	0.91	0.03	0.63	0.07	0.78	0.05	Sex
Lethargy	0.74	0.06	0.89	0.04	0.57	0.07	0.83	0.05	Trt, Sex
Standing	0.03	0.02	0.02	0.01	0.18	0.06	0.07	0.03	Sex
Touching	0.29	0.06	0.19	0.07	0.22	0.05	0.17	0.04	
Walking	0.12	0.04	0.06	0.03	0.20	0.06	0.04	0.02	Trt

a *Pig treatment levels: 1 mg/kg Poly(I:C) or Saline*.

b *Sex or Treatment (Trt) effect significant at P-value < 0.05*.

c *Probability estimates range from 0 to 1*.

**Figure 3:**
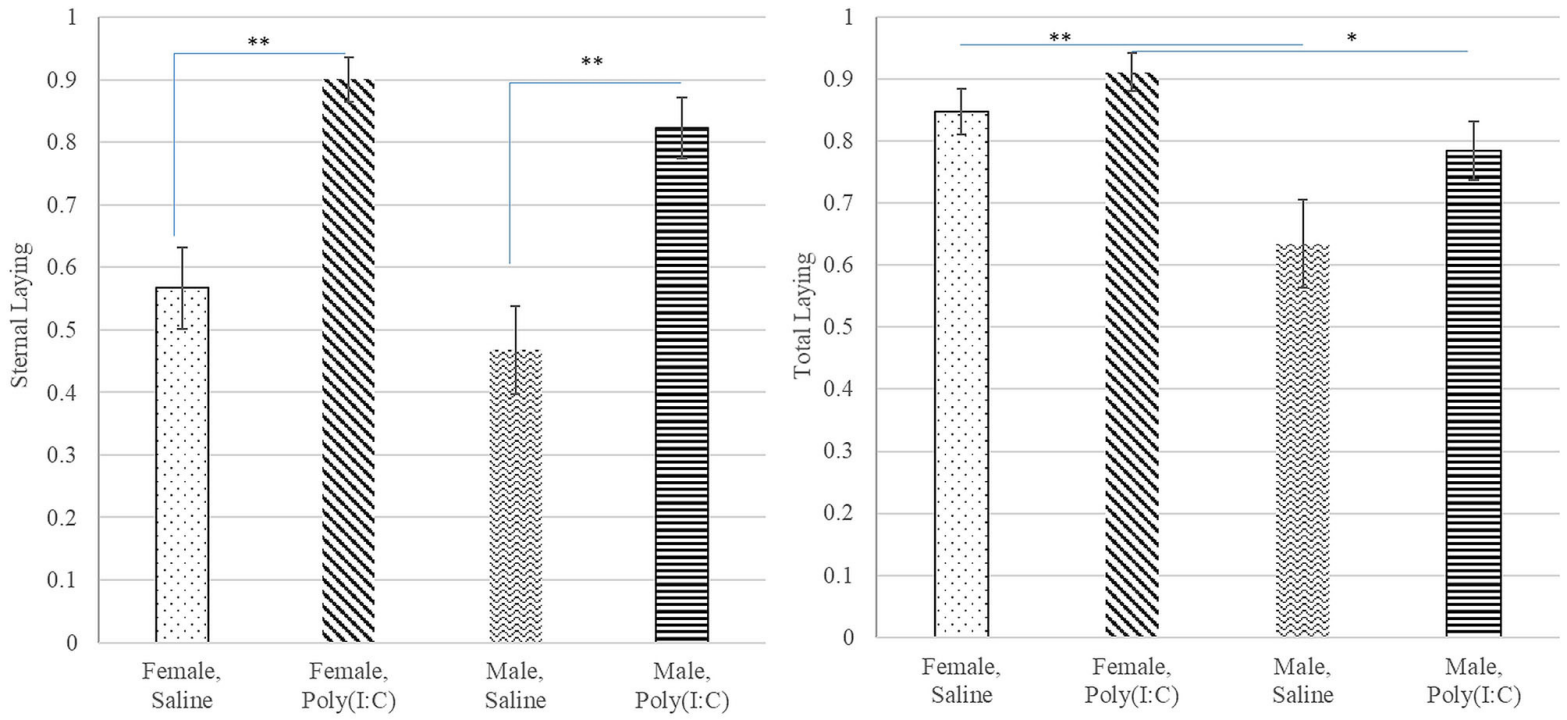
Probability of sternal **(left)** and total **(right)** laying behaviors (estimate and standard error of the mean) by Poly(I:C) treatment level (Saline or 1 mg/kg), and sex (Female or Male). **, *Difference between Poly(I:C)-sex groups significant at *P*-value < 0.005 and 0.05, respectively (*n* = 10).

The probability of total or overall laying (irrespective of position), lateral and sternal laying, and lethargy exhibited a significant treatment effect (Table 1). The pattern of incidence of overall laying followed that of sternal laying, which was the most prevalent laying position (Figure 3). Pigs receiving Poly(I:C) presented higher probability of laying than those receiving Saline (Table 2). The probability of lateral laying was opposite to sternal laying with lower probability among pigs that received Poly(I:C) relative to Saline injection. Also, the probability of laying was higher in females than males (Figure 3), with Poly(I:C)-treated pigs presenting higher probability of lethargy than Saline-treated pigs.

**Figure 4:**
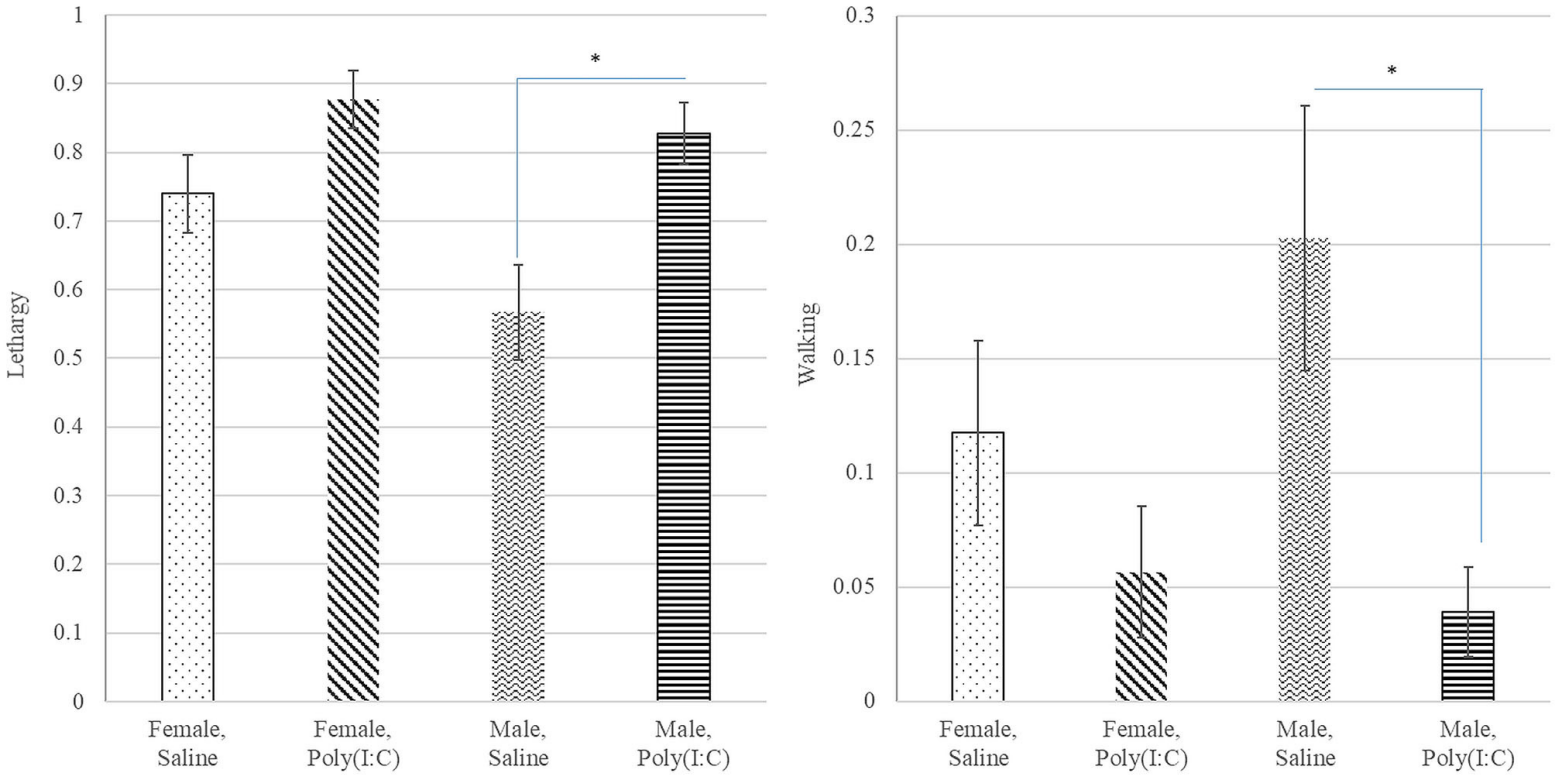
Probability of lethargy **(left)** and walking **(right)** behaviors (estimate and standard error of the mean) by Poly(I:C) treatment level (Saline or1 mg/kg) and sex (Female or Male). *Difference between treatment levels significant at *P*-value < 0.01 (*n* = 10).

Poly(I:C) treatment had a significant effect on the probability of walking (Table 1) with Saline-treated pigs presenting higher probability of walking than Poly(I:C)-treated pigs (Figure 4). The probabilities of standing and touching were higher among Saline-treated relative to Poly(I:C)-treated pigs (Table 2). The differences in the probabilities of walking and standing between Poly(I:C) and Saline groups were more marked in males than in females, whereas the differences in the probabilities of touching were more marked in females than males (Table 2). The probabilities of vomiting and panting were <0.03 in multiple treatment-sex groups, and therefore these results are not presented. We note that the probability of vomiting was over two-fold higher in Poly(I:C)-treated male pigs than in all other groups. The pattern of lethargy followed that of sternal laying with the probability of lethargy being higher in Poly(I:C)-treated relative to Saline-treated pigs (Figure 4).

### Experiment 3. Effects of Maternal PRRSV Activation, Poly(I:C) Immune Challenge, and Sex on Behaviors

The simultaneous effects of the first immune challenge elicited by PRRSV MIA during gestation, second immune challenge from the Poly(I:C) treatment, and sex on the behavior of pigs at PD 60 were evaluated in Experiment 3. A summary of the statistical significance (*P*-values) of the effects of MIA, Poly(I:C), sex and interactions across behaviors is presented in Table 3.

**Table 3:** Statistical significance of the effects of maternal immune activation elicited by the prenatal porcine reproductive and respiratory syndrome virus (PRRSV) challenge of the gilt, the pig immune treatment, sex, and interactions by behavior corresponding to Experiment 3.

**Behavior**	**MIA^a^**	**Sex**	**Pig** ** treatment^b^**	**MIA** ** by sex**	**MIA by** ** treatment**	**Treatment** ** by sex**	**MIA by treatment by sex**
Drinking/eating	0.646^3^	0.008	0.523	0.236	0.530	0.071	0.821
Lateral laying	0.184	0.399	0.014	0.509	0.090	0.857	0.017
Sternal laying	0.321	0.909	0.067	0.129	0.658	0.981	0.432
Total laying	0.796	0.374	0.833	0.070	0.235	0.469	0.179
Lethargy	0.809	0.397	0.835	0.054	0.323	0.352	0.238
Panting	0.442	0.304	0.114	0.572	0.522	0.254	0.031
Playing	0.050	0.048	0.496	0.002	0.563	0.185	0.789
Sitting	0.963	0.446	0.339	0.097	0.501	0.588	0.262
Standing	0.373	0.182	0.887	0.014	0.658	0.314	0.127
Touching	0.492	0.035	0.327	0.251	<0.0001	0.027	0.736
Walking	0.523	0.191	0.103	0.136	0.066	0.216	0.604

a *Maternal immune activation*.

b *Pig immune treatment levels: 1 mg/kg Poly(I:C) or Saline*.

*^c^P-value (n = 52 pigs)*.

A significant three-way interaction effect was detected for lateral laying and panting (Table 3). Complementing the previous findings, overall laying, lethargy, playing, sitting, and standing presented a MIA-by-sex interaction effect and touching presented a MIA by Poly(I:C) interaction effect.

Further confirmation of the effect of Poly(I:C) identified in Experiments 1 and 2 was achieved through the analytical partition of the effect of the second challenge within sex and MIA group. Likewise, additional insights into the effect of MIA were gained from the partitioning of this effect within second challenge and sex group. Table 4 presents the tests of the effect of second challenge within MIA and sex group, and Table 5 presents the corresponding tests of the effect of MIA within Poly(I:C) and sex group.

**Table 4:** Statistical significance of the effects of the pig immune treatment (Poly(I:C) or Saline) within maternal immune activation of gilts (porcine reproductive and respiratory syndrome virus or control groups) and sex group, by behavior corresponding to Experiment 3.

**Behavior**	**Control** ** female**	**PRRSV** ** female**	**Control** ** male**	**PRRSV** ** male**	**Control**	**PRRSV**	**Female**	**Male**
Drinking/eating	0.847^a^	0.493	0.100	0.162	0.333	0.991	0.500	0.038
Lateral laying	0.023	0.133	0.612	0.196	0.052	0.951	0.356	0.241
Sternal laying	0.201	0.985	0.626	0.473	0.213	0.611	0.394	0.388
Total laying	0.746	0.042	0.745	0.875	0.100	0.110	0.188	0.737
Lethargy	0.93	0.039	0.726	0.886	0.760	0.108	0.108	0.732
Panting	0.026	0.001	0.001	0.578	0.059	0.006	0.132	0.002
Playing	0.045	0.194	0.705	0.441	0.092	0.133	0.053	0.410
Sitting	0.676	0.994	0.834	0.260	0.857	0.136	0.761	0.398
Standing	0.711	0.194	0.826	0.353	0.921	0.63	0.387	0.597
Touching	0.097	0.001	0.001	0.201	0.001	0.001	0.109	0.124
Walking	0.399	0.006	0.970	0.177	0.540	0.004	0.008	0.307

a *P-value*.

**Table 5:** Statistical significance of the effects of maternal immune activation (MIA) of gilts (porcine reproductive and respiratory syndrome virus or control groups) within pig immune treatment (Poly(I:C) or Saline) and sex group, by behavior corresponding to Experiment 3.

**Behavior**	**Female,** ** saline**	**Female,** ** poly(I:C)**	**Male,** ** saline**	**Male,** ** poly(I:C)**	**Saline**	**Poly(I:C)**	**Female**	**Male**
Drinking/eating	0.359^a^	0.610	0.836	0.605	0.479	0.969	0.314	0.630
Lateral laying	0.913	0.015	0.229	0.463	0.467	0.075	0.139	0.290
Sternal laying	0.971	0.413	0.151	0.199	0.410	0.263	0.653	0.156
Total laying	0.987	0.043	0.588	0.524	0.746	0.413	0.257	0.489
Lethargy	0.879	0.057	0.564	0.486	0.812	0.484	0.245	0.457
Panting	0.512	0.138	0.649	0.381	0.917	0.725	0.644	0.824
Playing	0.014	0.020	0.934	0.808	0.110	0.059	0.003	0.920
Sitting	0.228	0.406	0.199	0.916	0.693	0.556	0.146	0.334
Standing	0.350	0.042	0.753	0.730	0.484	0.315	0.108	0.986
Touching	0.344	0.004	0.098	0.089	0.142	0.013	0.254	0.935
Walking	0.744	0.084	0.705	0.663	0.983	0.229	0.258	0.968

a *P-value*.

Statistically significant effects of the second challenge using Poly(I:C) on multiple behaviors were detected in female offspring within MIA group (Table 4). On the other hand, the effects of Poly(I:C) on males were restricted to pigs from Control gilts. The effects of Poly(I:C) on the probabilities of drinking or eating, lateral laying, overall laying, and playing were observed in specific MIA and sex groups.

The impact of MIA on the offspring behavior within second immune challenge group is characterized in Table 5. The lower probability of walking and higher probability of panting in Poly(I:C)-treated relative to Saline-treated pigs was consistent across Experiments 2 and 3. Among Saline-treated pigs, MIA had a significant effect (*P*-value < 0.014) on playing in females, and a borderline effect (*P*-value < 0.098) on touching in males. Many behaviors were affected by MIA among Poly(I:C)-treated females. The effect of PRRSV challenge on Poly(I:C)-treated females was significant (*P*-value < 0.05) for lateral and overall laying, lethargy, playing, standing, touching, and borderline significant (*P*-value < 0.084) for walking (Table 5). In general, MIA had a significant effect on playing among females irrespectively of second challenge, and on playing and touching among Poly(I:C)-treated-pigs irrespectively of sex. Tables 6, 7 summarize the estimated probability (and standard error) of individual behaviors within Poly(I:C) treatment-sex group for pigs from Control and PRRSV-challenged gilts, respectively.

**Table 6:** Probability of behavior estimates (Est) and standard error (SE) by group defined by the combination of immune treatment (Poly(I:C) or Saline) and sex for pigs from control gilts corresponding to Experiment 3.

	**Female**				**Male**				**Treatment**
	**Saline^a^**		**Poly(I:C)**		**Saline**		**Poly(I:C)**		**Effect**
**Behavior**	**Est**	**SE**	**Est**	**SE**	**Est**	**SE**	**Est**	**SE**	***P < 0.05*^b^**
Drinking/eating	0.04^3^^∧∧^	0.03	0.05	0.03	0.17^*^^∧∧^	0.06	0.05^*^	0.04	
Lateral laying	0.14^**^	0.07	0.02^**^	0.02	0.06	0.03	0.04	0.03	Female
Sternal laying	0.43	0.08	0.54	0.07	0.55	0.07	0.59	0.08	
Total laying	0.62	0.08	0.59	0.07	0.63	0.06	0.66	0.07	
Lethargy	0.59	0.08	0.60	0.07	0.63	0.07	0.66	0.08	
Panting	0.39^**^^∧∧∧^	0.18	0.22^**^^∧∧∧^	0.13	0.12^***^^∧∧∧^	0.08	0.48^***^^∧∧∧^	0.18	Female, male
Playing	0.27^**^	0.10	0.13^**^	0.06	0.15	0.06	0.13	0.06	Female
Sitting	0.03	0.02	0.04	0.02	0.06	0.03	0.05	0.03	
Standing	0.25	0.08	0.28	0.07	0.23	0.07	0.21	0.07	
Touching	0.44^*^	0.09	0.30^*^	0.07	0.47^***^	0.08	0.19^***^	0.06	Male
Walking	0.33^∧∧^	0.10	0.25	0.08	0.16^∧∧^	0.06	0.15	0.06	

a *Pig treatment levels: 1 mg/kg Poly(I:C) or Saline*.

b *Treatment effect significant (P-value < 0.05) in females or males from control gilts*.

*^c^Probability estimates range from 0 to 1*.

***, ** and * *Difference between Poly(I:C) treatment levels significant at P-value < 0.001, 0.05, and 0.1, respectively*.

∧∧∧ and ∧∧ *Difference between sexes significant at P -value < 0.005 and 0.05, respectively*.

**Table 7:** Probability of behavior estimates (Est) and standard error (SE) by group defined by the combination of immune treatment (Poly(I:C) or Saline) and sex for pigs from porcine reproductive and respiratory syndrome virus-challenged gilts corresponding to Experiment 3.

	**Female**				**Male**				**Treatment**
	**Saline^a^**		**Poly(I:C)**		**Saline**		**Poly(I:C)**		**Effect**
**Behavior**	**Est^b^**	**SE**	**Est**	**SE**	**Est**	**SE**	**Est**	**SE**	***P < 0.05*^c^**
Drinking/eating	0.01^^∧∧^^	0.01	0.03	0.02	0.19^^∧∧^^	0.07	0.08	0.05	
Lateral laying	0.15	0.12	0.32^^∧∧^^	0.18	0.2	0.14	0.09^^∧∧^^	0.07	
Sternal laying	0.43	0.12	0.42	0.11	0.33	0.11	0.39	0.11	
Total laying	0.61^**^	0.09	0.79^^**^^∧∧^^	0.06	0.57	0.10	0.58^^∧∧^^	0.09	Female
Lethargy	0.61	0.09	0.79^^∧∧^^	0.06	0.56	0.10	0.58^^∧∧^^	0.09	Female
Panting	0.23^***^	0.14	0.63^^***^^∧∧∧^^	0.18	0.19	0.13	0.25^^∧∧∧^^	0.15	Female, male
Playing	0.05^∧^	0.03	0.01^^∧∧^^	0.01	0.16^∧^	0.05	0.12^^∧∧^^	0.04	
Sitting	0.07	0.04	0.08	0.03	0.01	0.01	0.05	0.03	
Standing	0.14	0.08	0.07^^∧∧^^	0.04	0.19	0.10	0.26^^∧∧^^	0.11	
Touching	0.32^***^	0.09	0.67^^***^^∧∧∧^^	0.08	0.27	0.08	0.38^^∧∧∧^^	0.09	Female
Walking	0.27^*^	0.12	0.08^**^	0.04	0.2	0.10	0.11	0.06	

a *Pig treatment levels: 1 mg/kg Poly(I:C) or Saline*.

b *Probability of behavior estimates range from 0 to 1*.

c *Treatment effect significant (P-value < 0.05) in females or males from PRRSV-challenged gilts*.

***, **, and * *Difference between Poly(I:C) treatment levels significant at P-value < 0.001, 0.05, and 0.1, respectively*.

∧∧∧, ∧∧, and ∧ *Difference between sexes significant at P -value < 0.005, 0.05, and 0.06 respectively*.

Notably, the effect of Poly(I:C) was associated with a decrease in touching among pigs from Control gilts compared to pigs from PRRSV-challenged gilts (Table 6). The effects of Poly(I:C) treatment within sex among pigs from Control gilts were concordant with the previously described effects of Poly(I:C) within sex groups in Experiment 2. Consistent with Experiment 2, Poly(I:C)-treated pigs had higher sternal and lateral laying probabilities than Saline-treated pigs from Control gilts and sternal laying was more frequent than lateral laying across sexes (Table 6). Also consistent with results from Experiment 2, lateral laying presented a trend opposite to sternal laying, with higher probabilities in Saline-treated relative to Poly(I:C)-treated pigs.

The patterns of lethargy, touching, playing and walking probabilities between Poly(I:C) and Saline-treated pigs from Control gilts (Table 6) were in agreement with the patterns detected in Experiments 1 and 2. Also consistent with results from Experiment 2, Poly(I:C)-treated pigs from Control gilts presented higher probability of panting and lower probability of walking than Saline-treated pigs from Control gilts (Table 6). Additionally, Poly(I:C)-treated pigs presented lower probability of eating or drinking than Saline-treated pigs. The Poly(I:C) effects were sex-dependent with males presenting higher probabilities of panting, drinking, and eating than females, and females presented higher probability of walking than males.

The Poly(I:C) challenge had a significant effect on the probability of sternal laying among pigs from Control gilts, whereas the effect was minor among pigs from PRRSV-treated gilts across sexes (Tables 6, 7). The higher probability of sternal laying in Poly(I:C)- relative to Saline-treated pigs was more notable in females than males from Control gilts, and in males than females from PRRSV-challenged gilts. The probability of standing and walking were lower among pigs from PRRSV-treated relative to Control gilts across all Poly(I:C)-sex groups. Also, the impact of Poly(I:C) treatment on walking was observed in both sexes among the pigs from PRRSV-treated gilts (Table 7), and in females from Control gilts. The hindering effect of Poly(I:C) treatment on exertive behaviors such as walking and standing observed in pigs from Control gilts was augmented in females and extended to males that experienced MIA.

**Figure 5:**
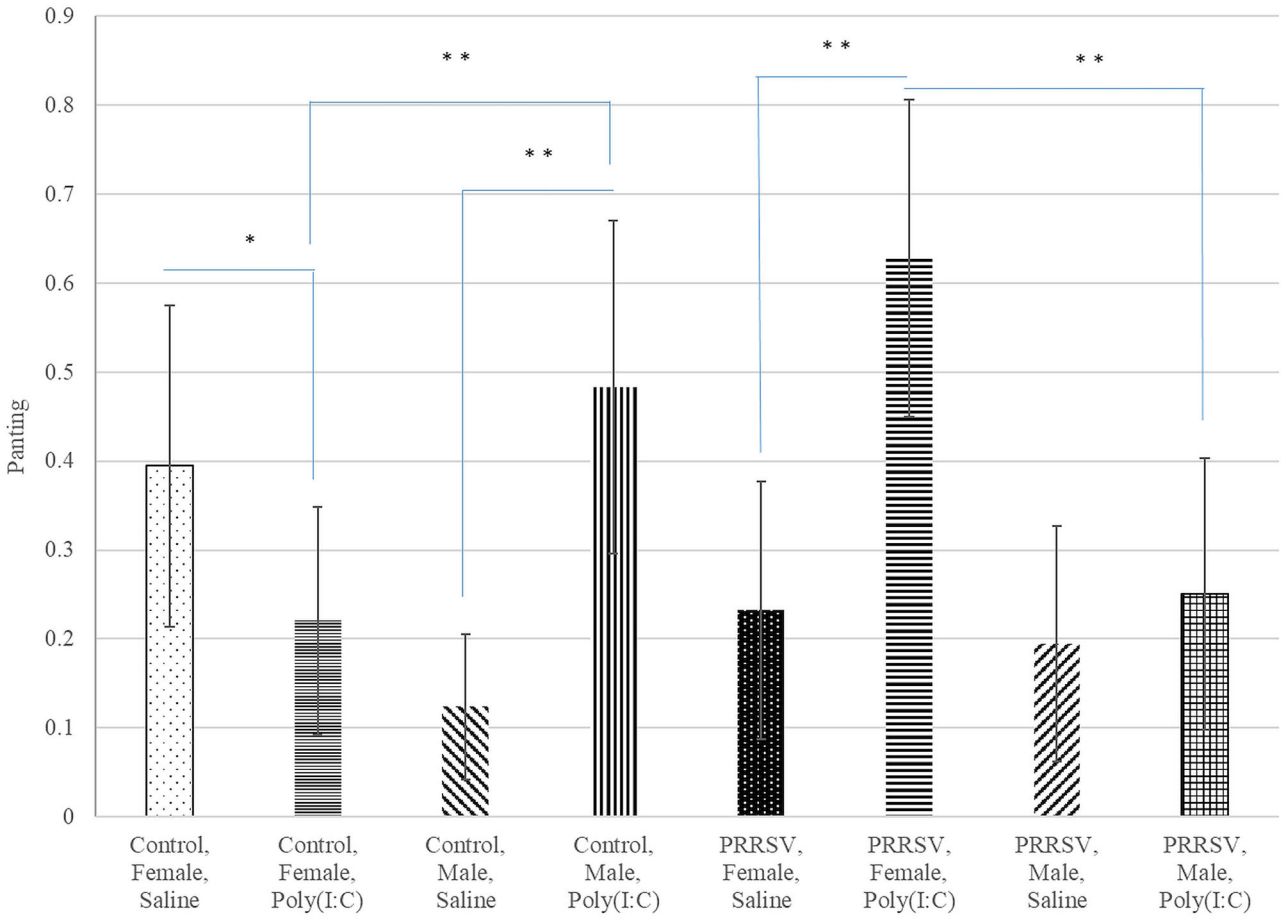
Probability of panting (logistic model estimate and standard error of the mean) across Poly(I:C) treatment level (Saline or 1 mg/kg), maternal immune activation (PRRSV-challenged or Control gilts), and sex (Female or Male). **, *Difference between treatment levels significant at *P*-value < 0.005 and *P*-value < 0.02, respectively (*n* = 52).

Poly(I:C) had similar effects on the probability of panting in males from Control gilts, and in all females regardless of MIA group (Figure 5). The significant effect of the interaction between Poly(I:C) treatment and sex on the probability of vomiting and panting arise from the male-only effect of Poly(I:C) on these sickness behaviors.

### Effects of Poly(I:C) and PRRSV Immune Challenges on the Body Temperature of Pigs and Gilts

The effect of Poly(I:C), MIA, sex, and all interactions on the PD 60 body temperature were evaluated in Experiments 2 and 3. Poly(I:C) treatment (1 mg/kg versus Saline) had a significant effect (*P*-value < 0.004) on the change in body temperature. The estimated change in body temperature after injection was 0.43°C (±0.11°C) for pigs treated with Poly(I:C) and−0.07°C (±0.12°C) for pigs treated with Saline.

A notable finding in Experiment 3 was that the change in the body temperature in response Poly(I:C) depended on the MIA group and sex of the pigs. The change in body temperature after Poly(I:C) injection was significantly higher (*P*-value < 0.02) among males from PRRSV-treated gilts (0.77°C ±0.11°C) than from Control gilts (0.24°C ±0.15°C). The change in body temperature after Poly(I:C) injection did not reach significance level yet follow the same pattern.

The effects of PRRSV treatment on the body temperature and feed refusal of the gilts were evaluated in Experiment 3. The average body temperature was 37.89°C (±0.03°C) and was not significantly different between PRRSV and Control gilts before GD 83 or after GD 92. The body temperature of PRRSV-treated gilts was significantly (*P*-value < 0.05) higher than Control gilts on GD 84-91 at, and borderline (*P*-value < 0.08) higher on GD 80-83 and GD 92-96. The highest difference in body temperature between PRRSV and Control gilts was 0.46°C (±0.23°C) on GD 87.

Feed refusal did not differ between PRRSV and Control gilt groups before GD 80 or after GD 103 (*P*-value > 0.05). The feed refused by PRRSV-treated gilts was significantly higher than Control gilts on GD 80-103 (*P*-value < 0.05), and borderline higher on GD 79 and 104 (*P*-value < 0.1). Control gilts did not refuse feed throughout the trial and the average feed refusal of PRRSV-treated gilts between GD 80 and GD 103 was 359.10g (±55.93 g).

## Discussion

A comprehensive characterization of the effects of PRRSV-elicited MIA, and of immune challenge elicited by a viral mimetic on the behavior of PD 60 female and male pigs was gained throughout three experiments. Experiment 1 aided in the identification of an effective Poly(I:C) protocol to elicit body temperature and behavior changes. Experiment 2 supported the characterization of the Poly(I:C) effect across sexes, and Experiment offered insights into the double hit paradigm combining PRRSV-elicited MIA and Poly(I:C) elicited second challenge.

### Experiment 1. Effects of Poly(I:C) Dose and Time on Behaviors Across Sexes

Pigs display behavioral changes in response to pathogens and pathogenic agents that can enhance the defense mechanisms (40). For example, behavior changes in response to PRRSV infection include motor inactivity or lower activity, lethargy, anorexia, and disruption in thermoregulation (41). These behavioral changes have been associated with changes in inflammatory cytokines (42). The effects of viral mimetic Poly(I:C) administered at a dose of 0.5 mg/kg body weight on 1-month-old pigs included changes in circulating interferon-alpha (26), and elevation in circulating interferon-2, and globulin (25). Results from our first experiment complement the previous reports, by evaluating a higher dose of Poly(I:C), and profiling the changes in sickness behaviors across time.

The present study complements the previous studies, evaluating a higher dose of Poly(I:C), and profiling the changes in behavior across multiple hours post-injection. The inactivity score of the pigs studied exhibited a significant treatment-by-sex-by-hour effect (*P*-value < 0.02, Figure 1). During the first hour after injection, all pigs that were administered 1 mg/kg of Poly(I:C) presented higher inactivity score than Saline-treated pigs whereas only males presented differential inactivity at half the dose.

The effect of Poly(I:C) lowering activity levels detected in the present study (Figure 1) is in agreement with reports of decreased movement in the pen, and increased lethargy among growing pigs challenged with Salmonella (43). Also consistent with our findings, male mice receiving an intraperitoneal injection of 12 mg/kg of Poly(I:C) exhibited lower horizontal and vertical activity, lower distance traveled, and lower speed of movements than mice treated with Saline (44). Our results suggest that the 0.5 mg/kg dose may be insufficient to elicit behavior changes in females. The higher dose required for a consistent behavior response in females is consistent with reports of the effect of an intraperitoneal injection of 12 mg/kg of Poly(I:C) on pubertal intact mice (45). The body temperature of Poly(I:C)-treated male mice was consistently higher than Saline-treated male mice during the first hour after injection, whereas an inconsistent pattern of temperature differences was observed in female mice.

The Poly(I:C) treatment had a significant effect (*P*-value < 0.019) on sickness behaviors characterized by vomiting, diarrhea, and shivering (Figure 2). Consistent with the effect on activity level, pigs receiving the 1.0 mg/kg and Saline doses presented the highest and lowest incidence of sickness behaviors, respectively. The prevalence of sickness behaviors associated with the intermediate Poly(I:C) dose studied was intermediate between the sickness behaviors observed at the extremes. The impact of Poly(I:C) on sickness behaviors observed in pigs was in agreement with the higher body temperature observed in pubertal male mice after Poly(I:C) injection relative to Saline-treated mice (45, 46). The results from Experiment 1 guided the study of 1.0 mg/kg of Poly(I:C) relative to Saline during the first hour after injection in the subsequent experiments.

### Effects of Poly(I:C) and PRRSV Immune Challenges on the Body Temperature of Pigs and Gilts

Ensuing the study of Poly(I:C) effects across time and doses, an investigation of the impact of this immune challenge on fever, a critical response to infection was undertaken. Fever constitutes a controlled rise in body temperature characterized by a net gain from increased heat production and decreased loss of temperature (42). Pigs from Experiments 2 and 3 that received 1 mg/kg of Poly(I:C) presented significantly higher body temperature after injection relative to pigs injected with Saline. A consistent effect of Poly(I:C) on body temperature was also reported in mice (45, 46). The increase in body temperature among Poly(I:C)-treated pigs relative to Saline-treated pigs is also in agreement with reports of fever in pigs experimentally infected with swine influenza (47). The febrile reaction to infection or other immune challenge is critical for the host to mount a protective inflammatory response (48). An increase in body temperature can support the host response to an immune challenge and therefore, behaviors that favor temperature maintenance may be more frequently displayed.

Parallel to the study of the febrile response of pigs to Poly(I:C), the response of gilts to PRRSV infection was also studied. In consideration of the extended response of gilts to PRRSV during gestation, the changes in body temperature and feed refusal were monitored in Experiment 3. The observed effect of PRRSV inoculation during the final third of gestation on the body temperature and feed refusal of the gilts in Experiment 3 was consistent with previous reports. In the present study, the body temperature of the PRRSV-treated group was significantly higher than the Control group on GDs 83-92. Feed refused by PRRSV-treated gilts was higher than Control gilts on GDs 80-103. Previous studies of gilts exposed to PRRSV on GD 76 also reported increases in body temperature and feed refusal within 2 weeks from infection (1, 49).

The comparison of temperature patterns between PRRSV-treated and Control gilts enabled the detection of a MIA-dependent change in body temperature in response to the second immune challenge in Experiment 3. Poly(I:C)-treated females and males from Control gilts presented higher elevation in body temperature than pigs treated with Saline. Pigs from PRRSV-treated gilts presented the same temperature pattern but the trend did not reach statistical significance. Our findings are consistent with reports that a mild LPS challenge during the neonatal period attenuated febrile responses to a similar immune challenge experienced later in life in female and male rats (50).

### Experiment 2. Effects of Poly(I:C) Immune Challenge and Sex on Behaviors

Following the characterization of the Poly(I:C) dose effect across time in Experiment 1, the analysis of the pig behavior patterns from Experiment 2 enabled a detailed evaluation of the effects of 1.0 mg/kg of Poly(I:C) relative to Saline within one hour after injection.

The significant effect of Poly(I:C) treatment on the probability of laying sternal, overall laying, and lethargy was characterized by higher incidence of these behaviors among the pigs in the Poly(I:C) group relative to those in the Saline group (Table 2, Figure 3). In agreement with our results, sternal laying has been associated with sickness behaviors in pigs (51, 52). The higher probability of laying in Poly(I:C)-treated pigs was consistent with the high frequency of lateral recumbency observed in weaned pigs after vaccination against a viral disease (53). Likewise, lethargy was observed in barrows treated with the immunostimulant LPS (54) or exposed to PRRSV (55).

The higher incidence of lethargy and vomiting behaviors in Poly(I:C)-treated males observed in Experiment 2 is consistent with results from Experiment 1, and is also in agreement with the effect of Poly(I:C) in raising body temperatures in pubertal intact male relative to female mice (45) and swine influenza in herds (47). The detection by the host immune system of pathogenic signals results in the stimulation of inflammatory mechanisms. Poly(I:C), LPS and viral infection trigger changes in peripheral inflammation and increase in pro-inflammatory cytokines in pigs and rodents (21, 25, 26). These responses interact with the brain to produce sickness behaviors.

Consistent with our results from Experiment 1, the odds of inactive behaviors such as laying in Experiment 2 were higher in Poly(I:C)- relative to Saline-treated pigs, whereas the odds of active behaviors such as touching or walking were higher in Saline-treated relative to Poly(I:C) pigs (Table 2). Also, the probability of these patterns are in agreement with reports of decreased movement in the pen, and increased lethargy among growing pigs challenged with Salmonella (43). The observed patterns are in agreement with lower probabilities of walking among 6-week old pigs directly inoculated with PRRSV relative to controls (41). This study also reported significantly lower eating and drinking among PRRSV-inoculated pigs relative to controls. Likewise, pigs infected with swine influenza displayed anorexia symptoms (47).

The difference between Poly(I:C)- and Saline-treated pigs in walking and standing probability was more extreme in male than in females, whereas the difference in touching rate was more extreme in females (Table 2). This result is consistent with reports that gilts were faster to touch a rope in the novel rope test, and faster to approach and touch a person in a human approach test than barrows (56). Similarly, increased social passiveness was observed in pigs within 24 hours of injection with the neuroinflammatory stimulant LPS (57). The effect of LPS was linked to changes in noradrenaline and serotonin levels in multiple brain regions, including the frontal cortex, which plays a role in selecting context-appropriate behaviors. Low cortical function was deemed responsible for the lower propensity of LPS-treated pigs to interact with their pen mates (57).

The interpretation of the Poly(I:C) effect on laying behaviors must consider the opposite patterns of sternal and lateral laying. The incidence of sternal laying was substantially higher than that of lateral laying. Laying could be an indicator of overall sickness status, or an indicator of relaxed and comfortable status, or an indicator of fatigue in response to high activity. The pigs in the Poly(I:C) group favored sternal laying and exhibited sickness behaviors and higher body temperature. Our study confirms reports that sternal laying can be associated with sickness (51, 52). Conversely, the higher incidence of lateral laying among pigs treated with Saline relative to Poly(I:C) could be associated with the higher walking probability in the same group, or with absence of sickness and overall wellness. Lateral laying has been associated with heat stress (58), and the higher walking probability in the Saline group may induce the pigs to subsequently lay laterally to eliminate heat. Pigs in the Poly(I:C) group exhibited higher body temperature and pigs with a fever would be more likely to exhibit behaviors that conserve heat, to maintain elevated body temperature.

### Experiment 3. Effects of Maternal PRRSV Activation, Poly(I:C) Immune Challenge, and Sex on Behaviors

The understanding accrued in Experiments 1 and 2, enabled us to use Poly(I:C) as a second immune challenge, subsequent in pigs exposed to MIA in Experiment 3. This capstone study compared pigs from gilts exposed to PRRSV during gestation relative to Control gilts, offering innovative insights into the prolonged effects of MIA and a second inflammatory agent on the behaviors of female and male pigs at PD 60. Prior to discussing the effect of MIA alone or interacting with Poly(I:C) and sex, we briefly focus the discussion on the results of pigs from Control gilts because this data subset enabled us to cross-validate the results from Experiment 2.

Among the behavior profiles consistent between experiments pigs treated with Poly(I:C) presented higher incidence of sickness behaviors and were less active than pigs treated with Saline (Table 6). Poly(I:C)-treated pigs had higher incidence of sternal laying and lethargy, and lower incidence of touching. Sternal laying was more frequent than lateral laying among Poly(I:C)-treated pigs from Control gilts, and the higher sternal laying probabilities were observed in both sexes. The higher probability of sternal laying among Poly(I:C) relative to Saline-treated pigs was more notable in females than in males. Also consistent across experiments, the probability of lateral laying presented a trend opposite to sternal laying in females, such that pig groups that have the highest lateral laying will also present the lowest sternal laying and vice versa. The probability of sternal laying was consistently higher than lateral laying in males across treatments, and higher in Poly(I:C) relative to Saline-treated males. This finding suggests that, sternal laying dominates in males and Poly(I:C) treated females whereas lateral and sternal laying were more commutable in Saline-treated females.

The results from Experiment 3 advance the understanding of the prolonged effects of PRRSV-elicited MIA, and of the inflammatory agent Poly(I:C) on the behaviors of female and male pigs at PD 60. For most behaviors a significant interaction between MIA and the second immune stressor was detected (Tables 4–7). This interaction could be characterized by discordant behavior changes in response to Poly(I:C) between MIA groups suggesting antagonistic first-second challenge effects. Alternatively, the interaction could be characterized by concordant behavior changes in response to Poly(I:C) between MIA groups (albeit in varying magnitude), suggesting synergistic first-second challenge effects. For fewer behaviors, the effect of Poly(I:C) on behavior was concordant in magnitude and direction across MIA groups, indicative of additive effects.

Discordant patterns of the effect of Poly(I:C) between MIA groups were observed in various behaviors including sternal and lateral laying, touching and walking across sexes (Tables 6, 7). Also, the increased lethargy after Poly(I:C) treatment was more marked in females from PRRSV-challenged gilts than other pig groups. The diminished effect of the second immune stressor on sternal laying of pigs from PRRSV-treated gilts showcases the prolonged effect of MIA on the response to a second challenge at PD 60, and suggests an antagonistic interaction between first and second immune challenges.

The lower probability of sternal laying in Poly(I:C)-treated pigs from PRRSV-challenged gilts relative to Control gilts was partially compensated by higher probability of lateral laying in males than females. An interesting finding is the higher probability of both lateral and sternal laying in Poly(I:C)-females relative to Saline-treated females from PRRSV-challenged gilts. The heightened laying and lethargy in females exposed to pre- and post-natal immune challenges than other pig groups suggests a synergistic interaction between first and second immune challenges for these behaviors.

Our results are consistent with studies of double-hit hypothesis in rodents. These studies surmise that prenatal immune challenge can augment (i.e., sensitization) or reduce (i.e., tolerance) the vulnerability of the brain and ensuing behavior disruption to subsequent challenges (20, 59, 60). As an illustration, a prenatal LPS challenge offered protection by lowering tissue loss and inflammatory response from hypoxia-ischemia brain stress in adult mice relative to non-LPS challenged mice. The inflammatory signaling pathways associated with the activation of nuclear factor-kappa beta and caspase-3 post-LPS challenge were implicated in the prenatal protection or preconditioning effects (20). Dysregulation of the nuclear factor-kappa b has been associated with behavioral deficits such as lower motor performance in mice and anxiety-like behaviors (61, 62). Likewise, ablation of caspase-3 was associated with reduced social interaction behaviors in male but not female mice (63).

In the present study, the differential effect of Poly(I:C) treatment across MIA and sex groups identified for behaviors such as laying and touching suggests that the first immune challenge can sensitize or protect from the effects of a second immune challenge on behaviors. In addition to the reversal of Poly(I:C) effect across MIA groups, pigs in the MIA group exhibited an enlargement of the effect of Poly(I:C) in panting and touching compared to pigs in the Control group suggesting that MIA sensitizes females to a second immune challenge. The effect of Poly(I:C) on panting is likely driven by the higher body temperature of pigs in this group, although tachypnea can also occur in conditions of acidosis that can be observed after LPS administration (64). Our results confirm suggestions that that sensitization or protection from disruptions associated with interactions among first and second challenge, respectively depend on factors such as sex (20). The effect of the interaction between first and second immune challenges on the relative propensity for lateral or sternal laying identified in females was not observed in males.

Among the concordant patterns of the effect of Poly(I:C) across MIA groups, drinking or eating, playing, and sitting probabilities associated with Poly(I:C)-sex groups were similar between MIA groups across sexes (Tables 6, 7). Additional concordant behavior patterns among MIA groups were identified on males alone for overall laying, lethargy, and panting. These finding suggest that, for some behaviors, the effect of pre- and post-natal challenges act in an orthogonal manner in males. The present study analyzed measurements recorded using synchronous and asynchronous (video-recorded) approaches. The consistency in findings across experiments suggests that both protocols enabled the detection of similar associations.

### Multi-Behavior Effects of Maternal Immune Activation, Second Challenge, and Sex

The integrative evaluation of the profiles across immune challenges and behaviors highlighted three main observations. The first observation relates to the three-way interaction between MIA, second immune challenge, and sex. For many behaviors, Poly(I:C) had a significant effect on females regardless of MIA, whereas Poly(I:C) appeared to affect mostly males from Control gilts. The significant effects of PRRSV challenge in the Poly(I:C) female group were less or not significant in either the Poly(I:C)-treated group nor among females. This result suggests a synergistic effect of maternal and Poly(I:C) challenges in females.

A second observation across behaviors relates to the two-way interaction between MIA and sex. The magnitude of the Poly(I:C) effect on the MIA-sex groups in many behaviors was consistent with the effect of Poly(I:C) on either MIA or sex groups separately. This result suggests an additive model of action of the first challenge and sex on the effect of the second immune challenge. A third broad observation relates to the effect of MIA alone, in pigs not exposed to a second immune challenge. Playing and touching probabilities were hindered in pigs exposed to MIA. Our findings are in agreements with reports of lower sociability and preference for social novelty of PD 14-28 pigs from gilts inoculated with PRRSV at GD 76 relative to pig from control gilts (1, 49, 65).

The behavior changes in response to a second immune challenge after MIA observed in the present study are in agreement with reports of the effect of MIA on related neuroimmune and physiological indicators (66). The combined effects of pre- and post-natal immune challenge on neuroendocrine axes (67), and neuroimmune responses (68, 69) are potential modulators of multiple behaviors studied. Moreover, the sex-dependent effects of pre- and post-natal immune challenges identified in the present study are in agreement with reports of differences between sexes in early life perturbations (70–72). Neonatal inflammation has been associated with deficits in memory tasks, motor function, social interaction and preferences and increased anxiety-like behaviors in female offspring (8, 73). Also, prenatal exposure to immune challenge was associated with lower juvenile social play, hyperactivity, anhedonia, and high hypothalamic-pituitary-adrenal axis responses to stress in males whereas females appeared unaffected (74–76).

### Implications

The present study advances the characterization and understanding of behavior changes across pre- and post-natal immune challenges in female and male 2-month-old pigs. The disruption of behaviors associated with a first immune challenge during gestation, and a second immune challenge can have deleterious effects on pig production and health. Poly(I:C) is a synthetic analog of a mismatched double-stranded RNA that binds to the toll-like receptor 3, and stimulates production of pro-inflammatory cytokines and associated phenotypes akin to RNA viral infections. The viral mimetic induces a transient immune response and therefore the molecular and organismal effects have a shorter duration than viral infections. Our study shows that (1) the single Poly(I:C) dose elicits observable behavior changes, and (2) the most extreme effects are observed within 1 h of injection.

Pre-injection baseline measurements allow for the pigs to serve as their own control group and aid in the control for confounding effect. The daily well-being checks did not identify pigs expressing outlier behaviors that could necessitate pre-injection measurements, and we wanted to minimize environmental disruptions. Therefore, measurements from the Saline group were considered as proxies for pre-injection measurements, covariates that could impact the behavior (i.e., gilt, pen, body weight) were included in the models used to analyze the data, and pigs were randomly assigned to the Poly(I:C) treatment groups.

The staggered overall experimental design used in the present study, encompassed independent and smaller experiments that enabled us to identify the safe dose of Poly(I:C) that triggered observable behavior changes, and the time after challenge when the changes would be detectable (Experiment 1), followed by validation of the findings (Experiment 2), and extension to the combined effect of MIA and Poly(I:C) later in life (Experiment 3). When analyzed separately, the size of the pilot and validation experiments (Experiments 1 and 2) hinder the power of the individual analyses. However, the distinct conditions of each experiment supported their separate analyses. The results from the larger sample size (*n* = 52) in Experiment 3 supported the previous findings. Additional studies recording information on the time course of behavioral changes, including baseline measurements and additional pigs, genotypes, and behaviors (e.g., feeder time, exposure to novel and familiar pigs and objects) will contribute to gain a more comprehensive understanding of the interplay among the factors studied.

In the present study, the higher probability of laying and lethargic behaviors among pigs exposed to MIA and to a Poly(I:C) immune challenge could increase the likelihood of lesions from other more active or aggressive pigs housed in the same pen. Also, the lower probability of walking and feeding or drinking behaviors among pigs from PRRSV-infected gilts injected with Poly(I:C) could hinder feed intake and growth. Touching and playing behaviors were affected by MIA and second immune challenges, and previous studies reported lower socialization and social novelty behavior on pigs exposed to MIA. The disruption of activity and social behaviors may interfere with the aptitude of MIA-exposed pigs to react to hazardous or unfamiliar conditions, or to detect social cues such as aggressive behaviors from pen mates (77). These conditions may augment the risk of MIA-exposed pigs for lesions, stress, hindered feeding and growth.

Our study explored the prolonged effects of neo- and post-natal diseases on the offspring behavior, through MIA alone or in combination with a second immune challenge. Behavior disruptions may in turn have potential impact on the health and performance of the offspring. Proactive strategies such as vaccination can reduce the risk of behavior modifications associated with MIA or double-hit. Likewise, housing and feeding systems, and pen assignment could be adapted to accommodate the distinct behavior of pigs exposed to MIA. Management practices could be proactively adopted to minimize the effects of pre- and post-natal immune challenges on pig behaviors that could potentially hinder pig performance and health.

## Data Availability Statement

The raw data supporting the conclusions of this article will be made available by the authors, without undue reservation.

## Ethics Statement

The animal study was reviewed and approved by The Institutional Animal Care and Use Committee at the University of Illinois approved the animal experiments.

## Author Contributions

SR-Z, RJ, and AA contributed conception and design of the study. SR-Z, RJ, and MV secured funding for the project activities. CB, LR, MC, AH, and JZ organized the animal experiments and collected the data. BS, HR, MV, and SR-Z planned and performed the statistical analysis. HR and SR-Z wrote the first draft of the manuscript. All authors contributed to manuscript revision, read and approved the submitted version.

## Conflict of Interest

The authors declare that the research was conducted in the absence of any commercial or financial relationships that could be construed as a potential conflict of interest.
